# A Soft Coral Natural Product, 11-Episinulariolide Acetate, Inhibits Gene Expression of Cyclooxygenase-2 and Interleukin-8 through Attenuation of Calcium Signaling

**DOI:** 10.3390/molecules18067023

**Published:** 2013-06-17

**Authors:** Wen-Li Hsu, Siou-Jin Chiu, Yao-Ting Tsai, Che-Mai Chang, Jaw-Yan Wang, Eric Terry Wang, Ming-Feng Hou, Chiung-Yao Huang, Jyh-Horng Sheu, Wei-Chiao Chang

**Affiliations:** 1 Department of Clinical Pharmacy, Taipei Medical University, Taipei 110, Taiwan; 2 Department of Physiology, College of Medicine, Kaohsiung Medical University, Kaohsiung 807, Taiwan; 3 Division of Gastroeintestinal and General Surgery, Department of Surgery, Kaohsiung Medical University Hospital, Kaohsiung Medical University, Kaohsiung 807, Taiwan; 4 Graduate Institute of Clinical Medicine, College of Medicine, Kaohsiung Medical University, Kaohsiung 807, Taiwan; 5 Cancer Center, Kaohsiung Medical University Hospital, Kaohsiung Medical University, Kaohsiung 807, Taiwan; 6 Department of Marine Biotechnology and Resources, National Sun Yat-sen University, Kaohsiung 804, Taiwan; 7 Graduate Institute of Natural Products, College of Pharmacy, Kaohsiung Medical University, Kaohsiung 807, Taiwan; 8 Department of Medical Research, China Medical University Hospital, China Medical University, Taichung 404, Taiwan; 9 Department of Pharmacy, Taipei Medical University-Wanfang Hospital, Taipei 116, Taiwan; 10 Master Program for Clinical Pharmacogenomics and Pharmacoproteomics, School of Pharmacy, Taipei Medical University, Taipei 110, Taiwan

**Keywords:** marine compound, EGF, COX-2, IL-8, calcium

## Abstract

Epidermal growth factor receptor (EGFR) is overexpressed in many types of cancer cells. EGFR-mediated signaling involves inflammatory gene expression including cyclooxygenase (COX)-2 and interleukin (IL)-8, and is associated with cancer pathogenesis. In a search of phytochemicals with anti-inflammatory activity, the COX-2 and IL-8 inhibitory activities of some marine compounds were examined. After screening these compounds 11-episinulariolide acetate (**1**) from soft coral exhibited the most potent activity. Reverse-transcription PCR; western blotting; ELISA and luciferase assays were used to test the effect of compound **1** on EGF-stimulated expressions of COX-2 and IL-8 in A431 human epidermoid carcinoma cells. After exposure to 10 μM of compound **1**, expression levels of COX-2 and IL-8 were reduced. In addition; intracellular Ca^2+^ increase and Ca^2+^-dependent transcription factor activation were blocked by compound **1**. Thus, compound **1** can potentially serve as a lead compound for targeting Ca^2+^ signaling-dependent inflammatory diseases.

## 1. Introduction

Epidermal growth factor receptor (EGFR), a member of the receptor tyrosine kinase superfamily that is overexpressed in different types of cancers, is associated with tumor malignancy [1]. In tumor tissues, high expression levels of cyclooxygenase (COX)-2 are detected [2,3]. In addition, the expression of COX-2 can be triggered by EGF or histamine in cancer cells [4,5]. Interleukin (IL)-8 is a pro-inflammatory chemokine that is activated in EGFR signaling [6,7,8,9]. IL-8 is known to induce matrix metalloproteinase (MMP)-9 release, which further regulates tumor cell proliferation, angiogenesis, invasion, and metastatic dissemination [10,11]. Both COX-2 and IL-8 can be mediated by intracellular Ca^2+^ concentration [5,12]. Ca^2+^ influx contributes to the mobilization of intracellular calcium and influences the expression of Ca^2+^-dependent transcription factors, such as the nuclear factor of activated T cells/nuclear factor IL-6 (NFAT/NF-IL6), cyclic AMP-responsive element (CRE) or nuclear factor (NF)-κB [5,13,14]. Therefore, calcium signaling plays an important role in EGF-mediated gene expression.

**Figure 1:**
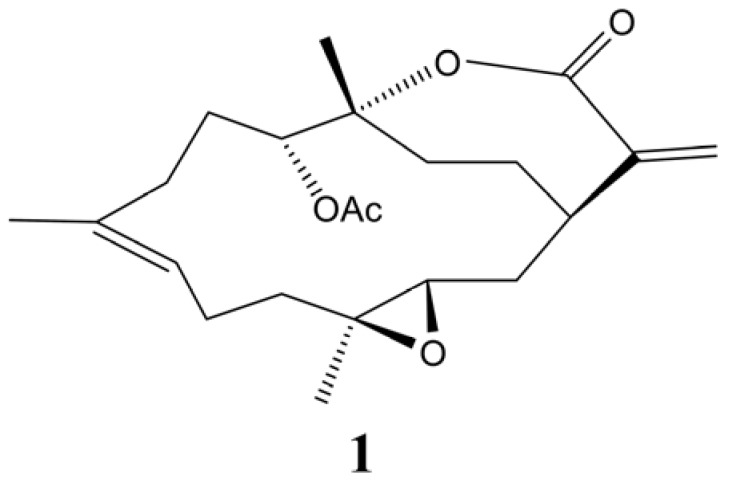
The chemical structure of the marine compound, 11-*epi*-sinulariolide acetate (**1**).

Soft corals of the genus *Sinularia* are known to produce 14-membered ring cembranoidal diterpenes as secondary metabolites [15]. In a previous chemical study of the soft coral *S. flexibilis*, collected from the coast of Pingtung, southern Taiwan, four cembranlides were isolated [16], including 11-episinulariolide acetate (**1**, Figure 1), which was first discovered by Kashman’s group [17]. In a recent chemical investigation of *S. flexibilis*, collected from the Dongsha Atoll (South China Sea), a large quantity (1,230 mg in 0.7 kg, dry wt) of compound **1** was found in this organism [18]. Previous biological activity studies revealed a weak-to-moderate cytotoxicity of compound **1** toward a variety of cancer cell lines [18,19] and a significant anti-inflammatory activity by inhibiting the accumulation of the pro-inflammatory inducible nitric oxide synthase (iNOS) and COX-2 proteins in lipopolysaccharide (LPS)-simulated RAW264.7 macrophage cells [18,20].

Increasing evidence indicates the anti-inflammatory and antitumor effects of natural compounds from marine organisms [21,22,23,24]. In this study, we examined whether marine compounds inhibit EGF-mediated COX-2 and IL-8 expressions in cancer cells. Our findings provide evidence to support the functional inhibitory activity of compound **1** on cytoplasmic calcium concentration COX-2 and IL-8 gene expressions. Thus, compound **1** could serve as a lead compound for targeting Ca^2+^ signaling-dependent inflammatory diseases and cancer metastasis potentially.

## 2. Results and Discussion

### 2.1. 11-Episinularidide (**1**) Inhibited COX-2 and IL-8 Expressions in Concentration-Dependent Manners

**Figure 2:**
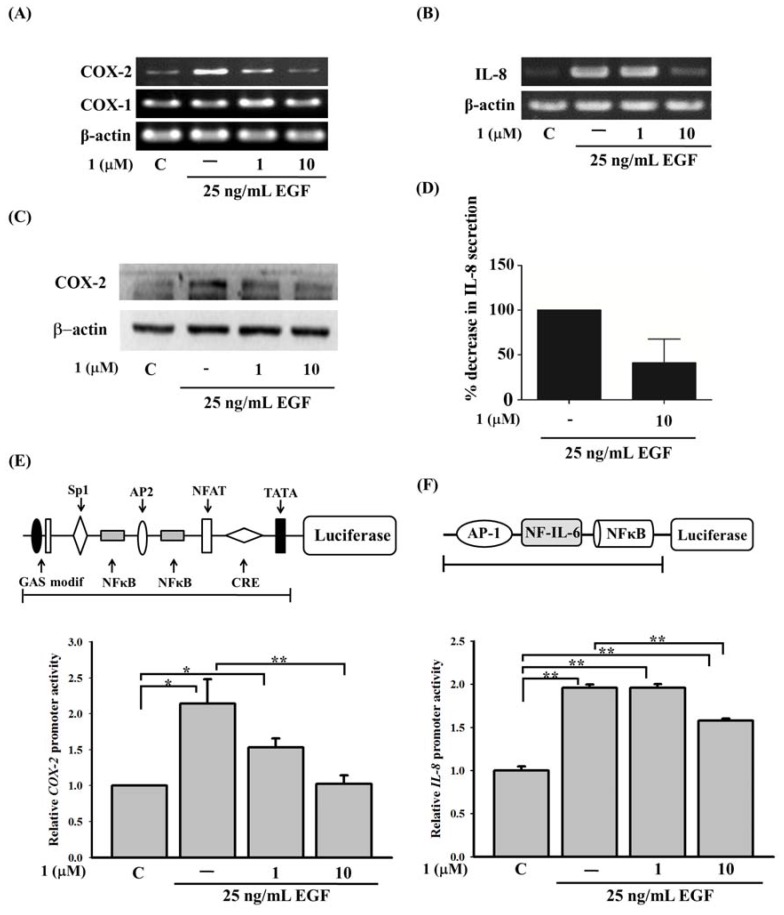
Effect of 11-episinularidide (**1**) on cyclooxygenase (COX)-2 and interleukin (IL)-8 expressions in A431 cells. Cells were pre-treated with 1 or 10 μM 11-episinularidide (**1)** for 30 min, and then stimulated with 25 ng/mL EGF for 3 h. Expressions of (**A**) COX-1, COX-2, and (**B**) IL-8 mRNA, which were detected from total RNA extracts of A431 cells, were measured using a RT-PCR. (**C**) Total cell lysates from A431 cells were prepared for Western blotting detecting COX-2 protein. (**D**) Supernatants from A431 cells were collected to measure IL-8 by ELISA. Cells were transiently transfected with 0.5 μg of (E) the COX-2 promoter plasmid, pXC 918, and (**F**) an IL-8 promoter plasmid for 24 h. Pretreatment with **1** was followed by the application of EGF, after which luciferase activity and total cell lysate concentrations were determined and normalized. Values for luciferase activity were calculated as the mean ± SEM. The statistical significance (* *p* < 0.05; ** *p* < 0.01) of differences between the results was determined using Student’s *t*-test.

To investigate the effect of compound **1** on EGF-mediated COX-2 and IL-8 expressions, A431 cells were used because of their abundance of EGFRs on the plasma membrane. EGF (25 ng/mL) was used to stimulate A431 cells. As shown in Figure 2, EGF induced gene activation of COX-2 and IL-8 (Figure 2A,B). The application of compound **1** resulted in the reduction of COX-2 (Figure 2A) and IL-8 (Figure 2B). Application of compound **1** has no effect on the expression of COX-1 or actin (Figure 2A). The protein levels of COX-2 and IL-8 also displayed similar results (Figure 2C,D). To further confirm these results, COX-2 and IL-8 promoter activities were analyzed by constructing a full-length promoter into the luciferase reporter plasmid, as described in the panels of (Figure 2E,F). With EGF stimulation, both COX-2 and IL-8 promoter activities were evoked. Application of compound **1** significantly inhibited EGF-induced COX-2 and IL-8 promoter activities (Figure 2E,F).

### 2.2. A SOCE Inhibitor, 2-APB, Inhibited COX-2 and IL-8 Expressions in a431 Cells

**Figure 3:**
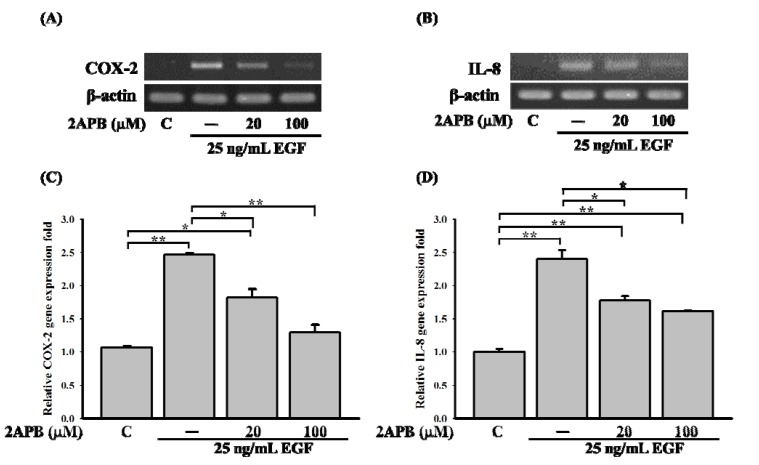
Effect of the SOCE inhibitor (2-APB) on cyclooxygenase (COX)-2 and interleukin (IL)-8 expressions in A431 cells. Cells were pretreated with SOCE inhibitor, 2-aminoethoxydipheny (2-APB), for 30 min, and then treated with 25 ng/mL of epidermal growth factor (EGF) for 3 h. Expressions of (**A**) COX-2 and (**B**) IL-8 were detected by an RT-PCR. Relative quantification in the RT-PCR of (**C**) COX-2 and (**D**) IL-8 was determined by normalizing to β-actin and a control (* *p* < 0.05; ** *p* < 0.01).

We next examined how compound **1** repressed EGF-mediated signal pathways, and how this compound affected COX-2 and IL-8 gene levels in A431 cells. Previous studies have indicated that COX-2 and IL-8 expressions can be regulated by Ca^2+^-dependent transcription factors [5,12,14]. Therefore, it is very likely that compound **1** could reduce COX-2 and IL-8 expressions by influencing calcium signaling. To determine the possible mechanism of compound **1** against COX-2 and IL-8 expressions, A431 cells were pretreated with the SOCE inhibitor, 2-aminoethoxydipheny (2-APB), and cells were then stimulated with EGF. As shown in Figure 3A,B, 2-APB reduced EGF-mediated COX-2 and IL-8 expressions in a concentration-dependent manner, from 2.45- to 1.25-fold (Figure 3C) and from 2.4- to 1.7-fold (Figure 3D) relative to the control, respectively. These results imply an important role of the store-operated calcium channel in EGF-mediated COX-2 and IL-8 expressions.

### 2.3. Effect of 11-Episinularidide (**1**) on EGF-Medicated Calcium Signaling in A431 Cells

**Figure 4:**
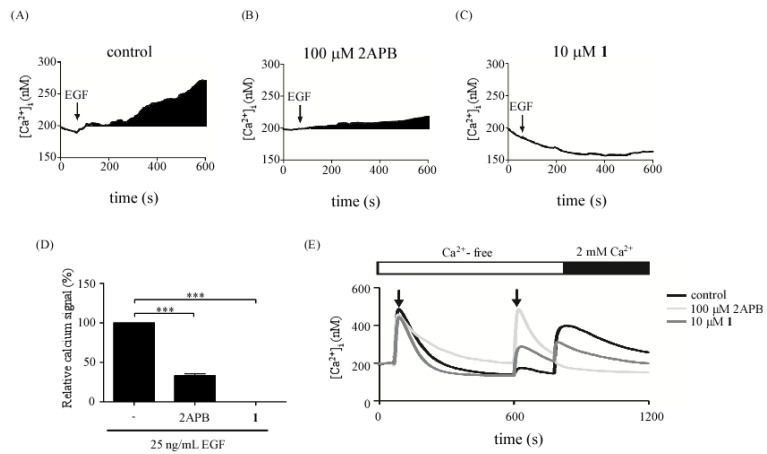
Effect of 11-episinularidide (**1**) on EGF-medicated calcium signaling in A431 cells. Time course of EGF-mediated cytoplasmic calcium signals was detected. Cells were stimulated with 25 ng/mL EGF in the control (**A**) and pretreated with 2-APB (B) and 10 μM **1** (**C**). The cells were loaded with Fluo-4-AM for Ca^2+^ detection and the Ca^2+^ response was due to a difference in the time constant utilized for averaging the signal. (**D**) The cytoplasmic Ca^2+^ signals were estimated by calculating the black areas under the Ca^2+^ curve in (**A**), (**B**) and (**C**). (**E**) Thapsigargin (2 μM TG) was applied to deplete the endoplasmic reticulum (ER) Ca^2+^ stores by inhibiting intracellular SERCA-type Ca^2+^ pumps in a Ca^2+^-free BSS solution as the first arrow indicated. The second arrow indicated the application of DMSO (control), 100 μM 2-APB or 10 μM **1** respectively. The extracellular Ca^2+^ concentration increased abruptly from 0 to 2 mM to trigger the SOC (store-operated channels) influx. The stepwise response was due to a difference in the time constant used for averaging the signal (*** *p* < 0.005).

According to previous results, compound **1** may affect EGF-medicated Ca^2+^ signaling, which in turn, inhibits EGF-mediated COX-2 and IL-8 expressions in A431 cells. To confirm this hypothesis, the effects of 2-APB or compound **1** in EGF-mediated calcium signaling were further investigated. Cells were pre-treated with 2-APB or compound **1** for 30 min and calcium signals were detected. EGF-medicated calcium (Figure 4A) was blocked by 2-APB (Figure 4B) from 100% to 31% (Figure 4D) and 10 μM compound **1** (Figure 4C) from 100% to 0% (Figure 4D), respectively. The results showed that inhibitory effects of compound **1** on calcium signals were similar to the effects of 2-APB. To further identify the effects of compound **1** on store-operated calcium entry, another calcium detection protocol was used. As shown in Figure 4E, a classical Ca^2+^ release was induced by thapsigargin. Compound **1** or 2-APB were added when the calcium store is empty. Interestingly, calcium release signals were evoked by 2-APB or compound **1**. The calcium release might be from mitochondria. We further re-introduced calcium into cells to evoke store-operated calcium influx. The store-operated calcium influx was reduced significantly by 2-APB, which is widely used for inhibition of inositol 1,4- 5-triphosphate (IP3) receptors, store-operated calcium channels and transient receptor potential (TRP) channels [25,26,27]. However, only a slight decrease of calcium influx was blocked by compound **1**. Thus, the target of compound **1** may not be store-operated calcium channel. Combined with the results in Figure 4D, the inhibitory effects of compound **1** in calcium signaling may be due to the attenuation of EGF-mediated signaling activation and intracellular calcium mobilization.

### 2.4. 11-Episinularidide (**1**) Inhibited EGF-Mediated COX-2 and IL-8 Expressions via Ca^2+^-Dependent Transcription Factors, NFAT and NF-κB Pathways

**Figure 5:**
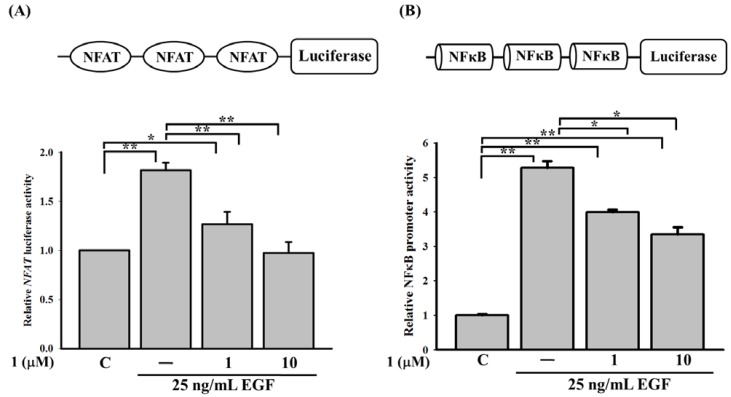
11-Episinularidide (**1**) affected the epidermal growth factor (EGF)-mediated activation of cyclooxygenase (COX)-2 and interleukin (IL)-8 promoters by the Ca^2+^-dependent transcription factors. Cells were transiently transfected with 0.5 μg plasmid containing (**A**) triple-NFAT binding sites or (**B**) triple-NF-κB binding sites. After incubation for 24 h, cells were pretreated with 1 or 10 μM of compound **1** before stimulation with the EGF at 25 ng/mL. After 3 h of treatment, the luciferase activity and cell lysate concentration of each group were determined and normalized (* *p* < 0.05; ** *p* < 0.01).

Intracellular Ca^2+^ mobilization can be altered by compound **1**. An analysis of the transcription factor-binding domain on the COX-2 promoter (Figure 2E) revealed the importance of Ca^2+^-dependent transcription factors, such as NFAT and NF-κB, which are major transcription factors in EGF-mediated COX-2 activation. Similarly, IL-8 promoter also contains an NF-κB-binding site. To further identify whether activation of NFAT and NF-κB was influenced by compound **1**, we used a luciferase assay to detect the inhibitory effect of compound **1** on NFAT- and NF-κB-binding sites. At concentrations of compound **1** from 1 to 10 μM, it reduced promoter activity of NFAT from 1.75- to 1.1-fold (Figure 5A) and that of NF-κB from 5.350- to 3.5-fold (Figure 5B) compared to the control. The results indicated that compound **1** may affect the promoter activities of NFAT and NF-κB by attenuating store-operated Ca^2+^ influx, thereby inhibiting EGF-mediated COX-2 and IL-8 expressions. From the literature, a Ca^2+^-dependent transcription factor, CREB protein, was shown to involve in EGF-medicated COX-2 gene expression [5]. We also found that compound **1** can influence the promoter activities of cyclin AMP-responsive element (CRE) (data not shown).

## 3. Experimental

### 3.1. Isolation of 11-Episinularidide (**1**)

The soft coral *S. flexibilis* was collected from Dongsha Atoll in March 2009. Compound **1** was obtainedby extraction of the organism with ethyl acetate and purification of the crude extract by column chromatography using a previously described method [18].

### 3.2. Cell Culture

The EGFR-rich A431 cell line was bought from the American Type Culture Collection (ATCC, Manassas, VA, USA). A431 cells were cultured in Dulbecco’s modified Eagle’s medium (DMEM; Invitrogen, Carlsbad, CA, USA) with 10% fetal bovine serum (FBS) (Invitrogen, Carlsbad, CA, USA) and 1% penicillin-streptomycin (Invitrogen) at 37 °C in 5% CO_2_. Before being treated with the drug, cells were grown in serum-free DMEM.

### 3.3. Reverse-Transcription Polymerase Chain Reaction (RT-PCR) and PCR

Total RNA was extracted from A431 cells with the Trizol reagent (Invitrogen). Reverse-transcriptase reactions required 1 μg RNA to synthesize complementary (c)DNA using an RT kit (Invitrogen). Incubation conditions included 10 min at 25 °C, 120 min at 37 °C, and 5 min at 85 °C. The resulting cDNAs were used to detect COX-1, COX-2, and IL-8 expression levels by the PCR. The following gene-specific primers were utilized: COX-1 (207 bp), forward primer: CCT CAT GTT TGC CTT CTT TGC and reverse primer: GGC GGG TAC ATT TCT CCA TC; human COX-2 (577 bp), forward primer: CAG CAA TTT GCC TGG TGA ATG ATT C and reverse primer: AGA CAG CGT AAA CTG CGC CTT T; human IL-8 (294 bp), forward primer: ACT TCC AAG CTG GCC GTG GCT CTC TTG GCA and reverse primer: TGA ATT CTC AGC CCT CTT CAA AAA CTT CTC; and human β-actin (145 bp), forward primer: ATC TCC TTC TGC ATC CTG TCG GCA AT and reverse primer: CAT GGA GTC CTG GCA TCC ACG AAA C. After denaturing the DNA at 94 °C for 5 min, 35 cycles of PCR were performed, with each cycle consisting of denaturation at 94 °C for 30 s, annealing at 58 °C for 1 min, and extension at 72 °C for 1 min. The PCR products were electrophoresed through the 2% agarose gel and visualized by ethidium bromide staining and UV transillumination.

### 3.4. Western Blotting

Total cell lysates (50 μg) were analyzed by SDS-PAGE on a 12.5% gel. After electro-blotting to nitrocellulose membrane, membranes were blocked with 5% nonfat dry milk for 1 h at room temperature. Membrane were washed with 0.1% PBST three times and then incubated with primary antibodies overnight at 4 °C. Antibodies against COX-2 (Abcam, Cambridge, MA, USA) and β-actin (Santa Cruz Biotechnology, Santa Cruz, CA, USA) were utilized as the primary antibodies. Mouse or rabbit IgG antibodies (Amersham Biosciences, Piscataway, NJ, USA) coupled to horseradish peroxidase were used as secondary antibodies. An enhanced chemiluminescence kit (Millipore Corp., Bedford, MA, USA) was used for detection.

### 3.5. ELISA

Human IL-8 secretion in cell-culture supernatants was assayed by enzyme-linked immunosorbent assay (ELASA) kit (eBioscience, San Diego, CA, USA) in accordance with manufacturer’s protocol.

### 3.6. DNA Transfection and Luciferase Assay

A431 cells were subcultured in six-well plates for 48 h. Then, cells were transfected with 0.5 μg of the COX-2 and IL-8 promoter plasmids in 1 mL of Opti-MEM medium (Invitrogen) containing 1 μL of Lipofectamine 2000 (Invitrogen) for 4 h. The next day, A431 cell were pretreated with **1**. Luciferase activity of the gene reporter was used dual-luciferase reporter assay kit (Promega, Madison, WI, USA) to measure multiples of gene activation.

### 3.7. Calcium Image

Cytoplasmic calcium signal was detected with stimulation by EGF or thapsigargin (TG) (Sigma-Aldrich, St Louis, MO, USA). Cells were incubated with 1 μM Fluo-4-AM (Molecular Probes, Eugene, OR, USA) at 37 °C for 20 min. Then cells were washed with BSS buffer (5.4 mM KCl, 5.5 mM D-glucose, 1 mM MgSO_4_, 130 mM NaCl, 20 mM HEPES pH 7.4, and 2 mM CaCl_2_). Ca^2+^ signals were detected and calculated based on the ratio of fluorescence intensities emitted upon excitation with consecutive 3-second pulses of 488-nm light at a resolution of 1376 × 1038 pixels by using an Olympus Cell^R IX81 fluorescence microscope (Olympus, Suite A Hicksville, NY, USA) equipped with an MT 20 illumination system (Olympus) and UPLanApo 10× objective lens. Intracellular Ca^2+^ concentration was estimated based on calibration curves as follows. A Ca^2+^ calibration curve was established by using a Ca^2+^ Calibration Buffer kit (Molecular Probes). Intracellular Ca^2+^ ([Ca^2+^]i) was calculated from Fluo-4 excited at 488 nm and was imaged by using an Olympus Cell^R IX81 fluorescence microscope and UPLanApo 10× objective lens at 20 °C. Fluo-4 signals were calibrated by measuring the fluorescence intensity from microcuvettes containing 10 mM K2EGTA (pH 7.20) buffered to various [Ca^2+^] levels. The following formula was used for Ca^2+^ concentration calculation: [Ca^2+^]I = KD × (F − Fmin/Fmax − F). Plotting the fluorescence intensity *versus* [Ca^2+^] yielded the calibration curve with the formula:

[Ca^2+^]_I_ = KD *(F − Fmin/Fmax − F)

where KD = 150.5 nM, F = Fluo-4 intensity, Fmax = 640, and Fmin = 21.7 for Fluo-4 [28].

## 4. Conclusions

**Figure 6:**
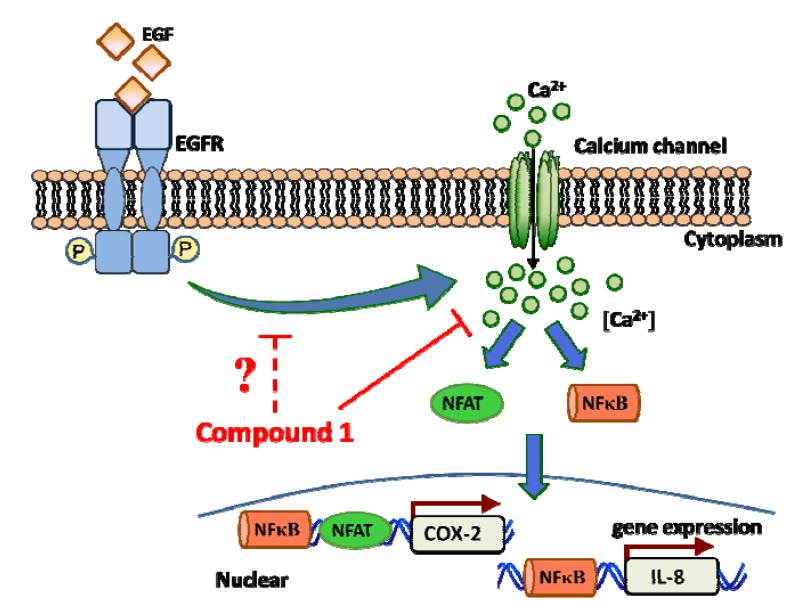
Schematic representation of compound 1 in the inhibition of cyclooxygenase (COX)-2 and interleukin (IL)-8 gene activities in A431 cells. EGF-mediated cytoplasmic calcium mobilization was attenuated by 11-episinularidide (**1**) that resulted in the inactivation of the Ca^2+^-dependent transcription factors including NFAT and nuclear factor NF-κB and suppression of IL-8 and COX-2 expression.

We identified the anti-inflammatory effects of compound **1** isolated from a soft coral. Compound **1** targeted EGF-mediated cytoplasmic calcium, which contributed to the inhibition of EGF-mediated COX-2 and IL-8 expressions. Ca^2+^ signaling has recently received greater attention in cancer research. Inhibition of store-operated Ca^2+^ influx may prevent the proliferation, metastasis, and angiogenesis of cancer cells [5,29,30] (Figure 6). Thus; compound **1** can potentially serve as a lead compound for targeting store-operated calcium signaling-dependent inflammatory diseases.
